# The PARP Enzyme Family and the Hallmarks of Cancer Part 2: Hallmarks Related to Cancer Host Interactions

**DOI:** 10.3390/cancers13092057

**Published:** 2021-04-24

**Authors:** Máté A. Demény, László Virág

**Affiliations:** 1 Department of Medical Chemistry, Faculty of Medicine, University of Debrecen, 4032 Debrecen, Hungary; 2 MTA-DE Cell Biology and Signaling Research Group, 4032 Debrecen, Hungary

**Keywords:** hallmarks of cancer, poly (ADP-ribose) polymerase, oncogenes, angiogenesis, inflammation, anticancer immunity, evasion of immune response, metastasis, invasion

## Abstract

**Simple Summary:**

Members of the poly (ADP-ribose)-polymerase (PARP) enzyme family regulate a broad range of cellular functions related to carcinogenesis, tumor growth, cell death, replicative immortality, and metabolism. In the companion paper (part 1) to this review, we covered how the 17 members of the PARP1 family affect these intrinsic cancer cell hallmarks. Here, we explore the PARP association of cancer hallmarks that derive from tissue-level reorganization in tumors and interactions of cancer cells with the tumor stroma. Thus, the focus of this review will be on the roles played by PARPs in tumor invasion, metastasis, anticancer immune responses, and tumor-associated inflammation. We present mechanisms that may enhance or weaken the therapeutic efficiency of PARP inhibitors and discuss the potential targeting of non-DNA dependent PARPs.

**Abstract:**

Poly (ADP-ribose) polymerases (PARPs) modify target proteins with a single ADP-ribose unit or with a poly (ADP-ribose) (PAR) polymer. PARP inhibitors (PARPis) recently became clinically available for the treatment of BRCA1/2 deficient tumors via the synthetic lethality paradigm. This personalized treatment primarily targets DNA damage-responsive PARPs (PARP1–3). However, the biological roles of PARP family member enzymes are broad; therefore, the effects of PARPis should be viewed in a much wider context, which includes complex effects on all known hallmarks of cancer. In the companion paper (part 1) to this review, we presented the fundamental roles of PARPs in intrinsic cancer cell hallmarks, such as uncontrolled proliferation, evasion of growth suppressors, cell death resistance, genome instability, replicative immortality, and reprogrammed metabolism. In the second part of this review, we present evidence linking PARPs to cancer-associated inflammation, anti-cancer immune response, invasion, and metastasis. A comprehensive overview of the roles of PARPs can facilitate the identification of novel cancer treatment opportunities and barriers limiting the efficacy of PARPi compounds.

## 1. Introduction

In two seminal papers, Hanahan and Weinberg proposed a set of quintessential traits that define cancer [1,2]. Six of these cancer hallmarks describe altered behaviors of transformed cells compared to their normal counterparts. According to these six hallmarks, cancer cells are characterized by genomic instability, sustained proliferative signaling, evasion of growth-suppressive signals, replicative immortality, altered metabolism, and resistance to cell death. In addition to these intrinsic cancer cell traits, four additional hallmarks/enabling characteristics define interactions between cancer tissue and the host organism [1]. This second set of hallmarks emphasize our view that cancer is a systemic disease, and cannot be adequately understood by scrutinizing only cancer cells. In other words, a tumor is closer to a complex organ than a lump of hyperproliferative cells. This second set of hallmarks arises from the derangement of signaling pathways arising from both the transformed cell and the homo- and heterotypic interactions between cancer cells, immune cells, and parenchymal or stromal cells. In the past decade, the interplay between the tumor and its microenvironment has become the prevailing direction of cancer research and the area from which the newest therapeutic modalities are emerging. The first regulatory body approval of a cancer immunotherapy in 2008; the approval of the first immune checkpoint inhibitor antibody by the FDA in 2011, followed by six others to date; the first approved chimeric antigen receptor (CAR) T-cell treatment in 2017; and hundreds of ongoing clinical trials all highlight promising new avenues for cancer treatment involving manipulation of the immune system’s interaction with transformed cells.

All hallmarks of cancer are closely intertwined with poly (ADP-ribose) polymerase (PARP)-mediated activities, and hallmarks related to cancer–host crosstalk are no exception. The PARP enzyme family, also known as ARTD (diphtheria toxin-like ADP-ribosyltransferase) enzymes [3], modify proteins with a single ADP-ribose residue (mono-ADP-ribosylation, MARylation) or poly(ADP-ribose) polymers (poly-ADP-ribosylation, PARylation) [4]. These enzymes are implicated in the regulation of the most fundamental cell functions, including genome maintenance, DNA repair, cell death, cell proliferation, and transcription [5,6,7,8,9,10,11]. In the first part of these twin reviews, we presented the fundamental roles of PARPs in intrinsic cancer cell hallmarks [12]. In the second part, we present evidence linking PARPs to cancer-associated inflammation, anti-cancer immune response, invasion of surrounding healthy tissues, and metastasis to remote organs.

## 2. Role of PARPs in Hallmarks Related to the Interplay between Cancer and Host

### 2.1. Angiogenesis

For continued growth and survival, a tumor must promote angiogenesis. In addition to the delivery of oxygen, nutrients, and growth factors, the neovasculature provides communication channels for cancerous cells to disseminate [13].

#### 2.1.1. Drivers of Angiogenesis in Tumors

**Figure 1:**
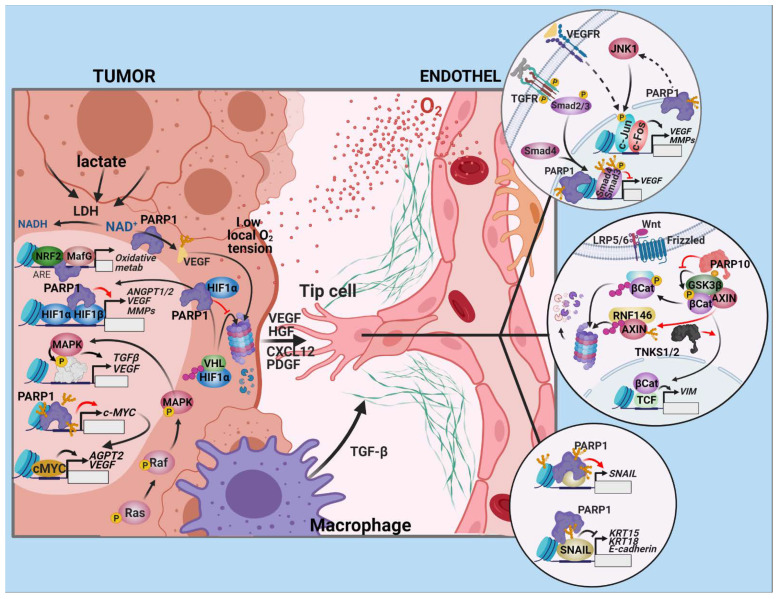
The contribution of PARPs to tumor angiogenesis. Angiogenesis is mainly promoted by low oxygen tension and altered metabolism in the interior of solid tumors. The response in cancer and the endothelial cells is primarily mediated by HIFs, which upregulate an array of angiogenesis-stimulating genes (e.g., VEGF, ANGPT2, CXCL12, HGF, PDGF-B). PARP-1 protects HIF1 and HIF2 from VHL-mediated ubiquitylation and proteasomal degradation, and also functions as a transcriptional co-activator for both. NRF2 stimulates mitochondrial oxygen consumption and enhances HIF1 activation by further reducing the intracellular O_2_ level. Although VEGF and VEGF2 are destabilized by PARylation, they can be preserved within the tumor because lactate, produced in excess by poorly oxygenized cancer cells with a rewired metabolism, reduces the NAD^+^ available for PARylation. PARP-1 also licenses the expression of c-MYC, another angiogenesis-related factor. The formation of new blood vessels begins with the EndoMT of a few endothelial cells in the vascular bed of the tumor site. The loss of apical-basal polarity and the adoption of a migratory phenotype, together with changes in molecular markers, are promoted by the TGF-β and WNT/β-catenin pathways (upper and middle callout circles). TNKS1/2 activate β-catenin by PARylating and targeting AXIN for proteasomal degradation. PARP-10 also stimulates β-catenin by MARylation-mediated inactivation of GSK3β, another negative regulator of WNT/β-catenin signaling. VEGF-stimulated endothelial cell migration depends on AP-1 signaling, and PARP-1 supports the AP-1 transcriptional response by activating JNK (upper callout circle). PARP-1 is involved at multiple levels in the positive regulation of SNAIL1, one of the most important transcription factors driving EndoMT (bottom callout circle). VIM: vimentin, KRT15/18: keratin 15/18.

A pivotal stimulus that drives the angiogenic process is the deregulation of oncogenic pathways. For example, the mutant RAS–RAF–MAP kinase signal transduction pathway can directly upregulate vascular endothelial growth factor (VEGF), transforming growth factor (TGF)-α/β, other growth factors, and cytokines that initiate vessel infiltration (Figure 1). In addition to the triggers inherent to the oncogenic process, angiogenesis is promoted by hypoxia, due to the increasing distance between capillaries and proliferating cancer cells as solid tumors grow [14]. Cells respond to hypoxia with a transcriptional program mediated by hypoxia-inducible factors (HIFs). The tumor suppressor protein, von Hippel–Lindau factor (VHL), is part of the Cul2–Rbx1–EloBC–VHL ubiquitin E3 ligase complex. Under normoxic conditions, VHL is responsible for the continuous removal of HIFs. In response to low oxygen tension or high levels of reactive oxygen species (ROS), this regulation is suspended and HIFs accumulate, translocate to the nucleus, dimerize, and upregulate their target genes. The most important angiogenic HIF target genes include VEGF-A, placenta growth factor (PGF), angiopoietin-2 (ANGPT2), chemokine (C–X–C motif) ligand (CXCL) 12 [15], hepatocyte growth factor (HGF) [16,17], and platelet-derived growth factor-B (PDGF-B) [18].

PARylation has been implicated in angiogenesis by numerous publications. PARP inhibition with GPI15427 blunts the endothelial response to angiogenic stimuli both in vitro and in vivo [19]. Defective growth factor-induced angiogenesis in PARP-1^−/−^ mice [19] and reduced angiogenesis marker expression of PARP1-depleted melanoma cells has provided further evidence for the role of PARP-1 in angiogenesis [20]. PARP-1 also contributes to HIF-1α accumulation in response to hypoxia, and this response is mediated by nitric oxide [21,22]. PARP1 physically interacts with HIF-1α, PARylates the transcription factor, and functions as a stability regulator and transcriptional coactivator in HIF-1α-dependent gene expression [23,24,25] (Figure 1). Accordingly, the expression of key HIF-1 target genes and the transcriptional activity of HIF-1 are compromised by PARP inhibition or in PARP-1^−/−^ cells [26]. PARP-1 also interacts with HIF2α and protects it from VHL-mediated ubiquitylation and degradation. Additionally, PARP-1 is required for the activation of HIF2 responsive genes and HIF-2α mRNA and protein expression [24]. PARP-1 inhibition reduced the vessel formation rate and vessel density count in subcutaneous HepG2 xenograft tumors in mice. This was associated with reduced expression of a number of tumor angiogenesis-related factors, including HIF2α, c-Myc, JunD, osteopontin, endoglin, VEGFR1, angiopoietin 2, MMP28, ADAM12, PDGF, and EGFR [27]. In a model of 12-O-tetradecanoylphorbol-13-acetate–induced skin carcinogenesis, arising papillomas exhibited decreased vessel density in PARP inhibitor-treated mice [26]. This effect on angiogenesis may be partially responsible for the somewhat paradoxical resistance of PARP-1^−/−^ mice against chemically-induced skin carcinogenesis. The differences in tumor vascularity likely account for decreased tumor size [28]. PARP inhibitors may dose-dependently reduce VEGF or bFGF-induced proliferation, migration, and tube formation in human umbilical vein endothelial cells (HUVEC) and the sprouting of rat aortic rings in vitro, suggesting that PARPs interfere with angiogenic signaling at a downstream point in the VEGF signaling pathway [27,29,30]. VEGF-induced endothelial migration is mediated primarily by the AP-1 transcription factor [31]. PARP-1 promotes the AP-1 transcriptional response by activating c-Jun N-terminal kinase (JNK), and AP-1 signaling is reduced in a PARP-1^−/−^ background [32,33]. Lactate, a prominent product of the rewired tumor metabolism, reduces PAR-synthesis by decreasing intracellular NAD^+^ content in the cells of the tumor microenvironment through LDH [34]. Exogenous lactate upregulates VEGF and VEGFR2 in HUVEC cells, and increases VEGF angiogenic potency by reducing its PARylation [35].

PARP-1 represses CXCL12 expression by changing the epigenetic control of its promoter. In PARP-1^−/−^ mouse embryonic fibroblasts, the expression of the 10–11 translocation (Tet) demethylases is increased, and the CXCL12 gene becomes demethylated and highly transcribed [36]. Another transcription factor, through which PARP-1 promotes angiogenesis, is nuclear factor erythroid 2—related factor 2 (NRF2). NRF2 and HIF signaling are intricately connected. NRF2 is activated by increased ROS in the tumor microenvironment. NRF2 silencing suppresses HIF1 activation, tumor vascularization, and growth under hypoxic conditions. Thus, reduced mitochondrial oxygen consumption in NRF2-inhibited cells prevents the stabilization of HIF1 [37]. PARP-1 stimulates the DNA-binding of NRF2 and the expression of its target genes upon binding to transcription factor MafG, the heterodimerization partner of NRF2, and its antioxidant response element (ARE) [38].

The function of c-MYC is also necessary for the angiogenic switch required for tumor progression. Expression of VEGF, angiopoietin 2, and other angiogenic factors requires c-MYC. Thus, vasculogenic defects develop in c-MYC^−/−^ mice embryos and ES cell xenografts [39]. PARP-1 promotes the transcription of c-MYC by PARylating the chromatin over its promoter when serum-starved cells are allowed to reenter the cell cycle [40].

#### 2.1.2. The Endothelial Response

During angiogenesis, endothelial cells (ECs) reenter the cell cycle, begin to divide, and migrate into the surrounding tissues. ECs then organize into hollow tubes, reestablish stable contacts with the extracellular matrix (ECM) with the help of integrins, and evolve into a mature network of blood vessels. A precondition of angiogenic sprouting is a partial endothelial-to-mesenchymal transition (EndoMT) of the endothelial cells, similar to the process of epithelial-to-mesenchymal transition (EMT). These transitions entail the loss of apical–basal polarity, degradation of the ECM, and adoption of a migratory phenotype, which involves the shedding of endothelial molecular markers (CD31, vascular endothelial (VE)-cadherin), and the acquisition of mesenchymal markers (smooth muscle actin (SMA), vimentin). The TGF-β and the Wnt/β–catenin pathways are the most important regulators of EndoMT. PARPs impact both of these pathways. Tankyrases 1 and 2 (TNKS1/2; also known as ARTD5 and ARTD6, PARP5a and PARP5b) activate β-catenin by poly (ADP)ribosylating AXIN, the scaffold protein in the β-catenin destruction complex, promoting its ubiquitylation by RNF146 E3 ubiquitin-ligase and thereby targeting it for proteasomal degradation. TNKS inhibition is known to stabilize AXIN and inhibit Wnt signaling [41]. PARP-10 has been shown to MARylate, and thus, inactivate the kinase GSK3β. GSK3β is another component of the β-catenin destruction complex that prevents the accumulation, nuclear translocation, and transcriptional activity of β-catenin [42]. A notable mesenchymal marker upregulated by β-catenin/TCF is vimentin [43]. PARP inhibition leads to the downregulation of vimentin, up-regulation of VE-cadherin, and the reversal of EndoMT in endothelial cells. Thus, PARP inhibition prevents malignant melanoma cells from developing vasculogenic mimicry [44]. SNAIL1, SNAIL2, and their downstream target, ZEB1, are major EMT- and EndoMT-inducing transcription factors. SNAILs repress E- and VE-cadherin, while they induce SMA and vimentin. PARP-1 is involved in the regulation of SNAIL expression at multiple levels. PARP-1 interacts with the SNAIL1 promoter and induces its transcription via PI3K-integrin-linked kinase (ILK)-Akt signaling. This signaling pathway integrates cues from the surrounding ECM. PARP-1 also PARylates and interacts with SNAIL1 [45]. Both PARP-1 knockdown and PARP inhibition lead to the accelerated degradation of SNAIL1 [46]. In contrast, PARylation also plays a pro-angiogenic role; Smad function is negatively regulated by PARP-1. PARP1 is assisted by PARP-2 in this pro-angiogenic role, and is positively regulated by PARG during the course of TGF-β signaling [47,48].

#### 2.1.3. Open Questions and Prospects

Altogether, PARPs appear to be proangiogenic by many accounts. However, the extent to which the antiangiogenic effects of PARPis contribute to the anticancer effect of these drugs is unclear. This effect is unlikely to result in a dramatic reduction in tumor vascularization, although it has not been investigated systematically. Nonetheless, numerous clinical trials are underway to test the efficacy of PARPi in combination with angiogenesis inhibitors.

### 2.2. Invasion and Metastasis

The development of malignant tumors culminates in the most dreadful changes from the perspective of the patient: Invasive growth and dissemination. Over 90% of cancer-related deaths are due to metastatic disease. The process of metastasis incorporates a number of steps, including invasion, intravasation, transport in the circulation, extravasation, survival at the metastatic site, micrometastasis formation, and colonization [49]. The current understanding of the molecular events in invasion and metastasis was obtained from malignancies of epithelial origin. The process begins with a phenotypic alteration within individual cells or tumor cell collectives at the invasive front of the primary tumor. EMT is an epigenetically directed program that does not require the acquisition of mutations and is necessary for the development of embryonic germ layers during gastrulation and neural crest formation [50,51]. EMT regulatory pathways and the main regulators of this pathway were briefly introduced in the previous section. Molecular interactions described above for EndoMT (e.g., PARP1′s involvement in SNAIL1/2 expression and signaling, tankyrase 1 and 2-mediated activation of β-catenin by AXIN PARylation, PARP-10-catalyzed MARylation, and inactivation of GSK3β kinase) are also relevant for EMT. Moreover, Yes-associated protein (YAP) is a proto-oncogene transcription factor elevated in human cancers that promotes metastasis [52,53]. YAP is inhibited by the Hippo pathway, which mediates cell–cell contact inhibition and tissue growth control [54]. The angiomotin (AMOT) protein family are suppressors of YAP activity [55]. TNKSs interact with AMOTs and promote their degradation through RNF146, similar to the regulation of β-catenin [56].

**Figure 2:**
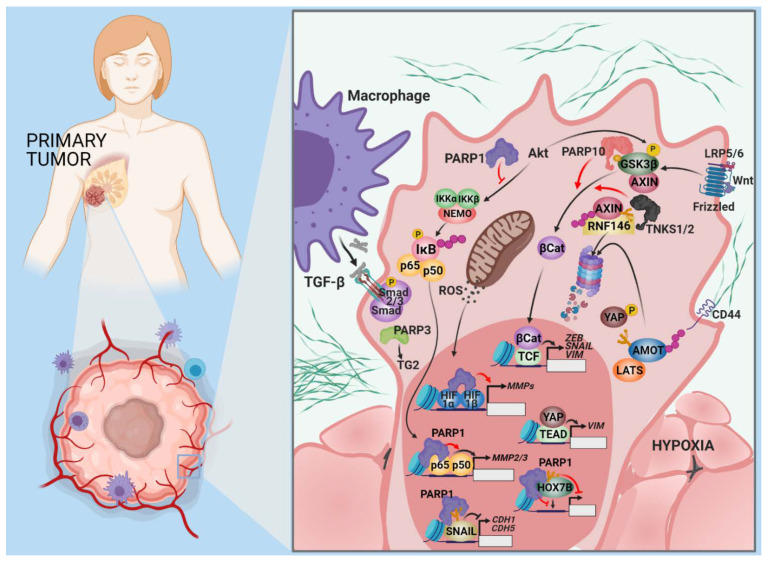
The association of PARPs with tumor cell invasion. Tumor cell invasion begins with EMT, a phenotypic transformation of the tumor cells. PARPs can have opposing effects on EMT. The Hippo pathway suppresses metastasis initiation by regulating the transcription factor, YAP, through the AMOT protein family members. TNKS1 and TNKS2 inactivate the AMOT proteins in a manner similar to their effect on AXIN, allowing the accumulation, nuclear translocation, and transcriptional engagement of YAP. PARP-1 interacts with HOXB7, another EMT-promoting transcription factor, and reduces its transcriptional activity. PARylation of Smad proteins by PARP-1 and -2 hinders TGF-β signaling. The PARPs are also involved in restructuring the ECM, which is necessary for the growth and dissociation of a tumor. Upregulation of PARP-1 has been connected to the transcriptional induction of MMP2 and 9 and the downregulation of TIMP. These effects are probably achieved through the stimulation of NF-κB transcription factors. PARP-1 is involved at multiple levels in the positive regulation of SNAIL1, one of the most important transcription factors driving dedifferentiation associated with EMT.

PARPs also exert EMT-suppressing effects. The homeobox transcription factor, HOXB7, is upregulated in mammary carcinomas, and HOXB7 expression confers EMT-like phenotypical features in breast cancer cells [57]. HOXB7 interacts with PARP-1 and undergoes PARylation, resulting in reduced transcriptional activity [58]. PARylation of Smad proteins by PARP-1 and PARP-2 and dePARylation by PARG are key negative and positive regulatory events controlling the strength and duration of Smad-mediated transcription during TGF-β signaling [47,48]. Inactivation of PARP-1 in mice with transgenic prostate adenocarcinoma resulted in higher TGF-β-driven Smad signaling. This increased Smad signaling correlated with the induction of EMT, loss of E-cadherin, and upregulation of N-cadherin and ZEB-1 [59] (Figure 2). The complex interactions between PARPs and EMT-regulating pathways are exemplified by the role of PARP-3 in TGF-β and ROS-dependent EMT, as well as the stem-like feature expression in human mammary epithelial and breast cancer cells. PARP-3 expression is higher in breast cancer cells with a mesenchymal phenotype and correlates with the expression of vimentin, a mesenchymal marker. PARP3 inversely correlates with the epithelial marker, E-cadherin. ROS generated in response to TGF-β activation induces PARP-3 expression, which in turn upregulates transglutaminase 2, a known regulator of the SNAIL and E-cadherin axis during EMT [60].

The genetic plasticity underlying EMT facilitates adaptation to cytotoxic or targeted therapies, and may result in acquired drug resistance [61]. Genetic plasticity and PARP’s effects on EMT-promoting pathways translates into a clinical response to PARP-inhibitor therapy that is difficult to predict. A single dose of olaparib (a PARP inhibitor) quickly upregulates EMT markers in treatment-naïve BRCA1/2-mutant breast tumors. Thus, EMT upregulation is one potential mechanism that may lead to treatment resistance during PARPi therapy [62].

At the invasive front, matrix metalloproteinases (MMPs) play a major role in tumor cell escape from primary carcinomas by reorganizing the ECM components and basement membrane [63]. Hepatocyte growth factor (HGF) expression plays a role in the invasiveness of ovarian tumors in patients with hepatic metastases of colorectal cancer relapsing after neoadjuvant chemotherapy. HGF also induces the acquisition of CSC (cancer stem cell) characteristics in pancreatic cancer [64,65,66]. HGF promotes the upregulation of PARP-1 connected to increased expression of MMP-2 [67]. PARP-1 is also a direct transcriptional activator of MMP-9 [68] (Figure 2). PARPi treatment results in decreased MMP-2 and MMP-9 expression and the induction of the tissue inhibitor of metalloproteinases (TIMPs) [69,70,71]. The NF-κB pathway, which is positively influenced by PARP-1, stimulates MMP expression [72]. Although RNAi-mediated silencing of PARG in colon cancer cells increases the level of phosphorylated Akt, PARG silencing still inhibits metastatic potential due to decreased expression of NF-κB, MMP-2, and MMP-9 [73]. An epistatic effect exist between the PARP-1 rs1136410 SNV that gives rise to a Val762Ala sequence variant with reduced enzymatic activity, and the rs243865 SNV of the MMP-2 gene promoter, which is accompanied by an increased risk of lymph node metastasis in gastric cancer [74,75].

**Figure 3:**
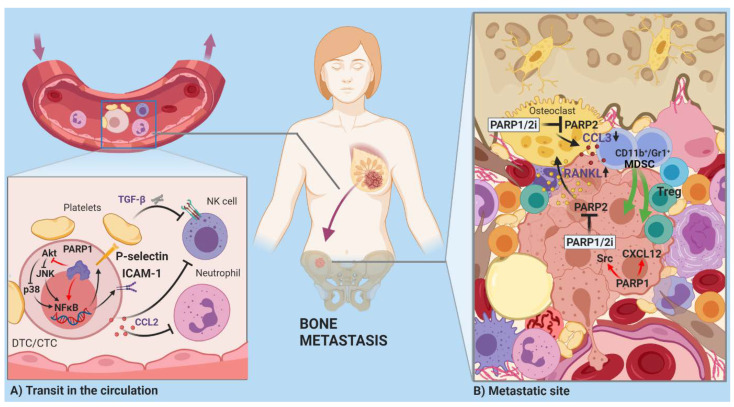
PARPs and metastasis. (**A**) In circulating disseminated tumor cells (DTCs), PARylation is involved in the downregulation of adhesion molecules (e.g., ICAM1, P-selectins), which establish contacts between tumor cells and platelets, offering protection from NK cells and granulocytes. PARP-1 is also instrumental in the expression of CCL2, a cytokine that educates granulocytes to not recognize DTCs. (**B**) DTCs lodged at metastatic sites must rely on cell death avoidance mechanisms to resist the hostile reactive stroma and attacks from innate immune cells (see also [12] and Figure 4). DTCs have a predilection for environments rich in survival factors characteristic of the primary tumor site. PARP-1 can reinforce Src signaling in the metastases of primary breast tumors, in which the Src pathway has been activated by estrogen. PARP-1 shuts down the CXCL12 promoter, which negatively affects the metastatic potential of CXCL12-dependent breast tumors in bone marrow. The complex interactions between tumor cells and stromal cells at a metastatic site can profoundly affect the outcome of chemotherapy. This is the case in BRCA1/2-negative breast cancer with a bone marrow metastasis, where the inhibition of PARP-1 is therapeutically beneficial, but the simultaneous inhibition of PARP-2 regulates CCL3 in osteoclasts, leading to colonization of the bone marrow, with immune-suppressive T cells facilitating the survival of the tumor cells.

Time spent in circulation is a risky period for circulating or disseminated tumor cells (CTCs/DTCs). Circulation exposes tumor cells to shear forces and immune system attack [49]. Cancer cells cover themselves with platelets to avoid harmful interactions with NK cells and neutrophil granulocytes [76,77]. TGF-β secreted by platelets transmits suppressive signals to immune cells and maintains EMT in the traveling tumor cells. PARylation may be involved in the expression of cell adhesion molecules [78] that mediate interactions with platelets (Figure 3A). Silencing of PARG in CT26 colorectal carcinoma cells increases the phosphorylation of AKT and decreases the expression of NF-κB and the cell adhesion molecules, ICAM-1 and P-selectin [79]. The CTCs/DTCs educate neutrophils and monocytes in the vasculature with C–C motif chemokine 2 (CCL2) to enhance vascular permeability, facilitating transmigration after homing at a potential metastatic site. PARP-1 induces CCL2 expression through NF-κB p65 [80] (Figure 3A).

A fortunate bottleneck exists in the invasion–metastasis cascade at the tumor initiation stage once the CTCs/DTCs reach the metastatic site. Most cells succumb due to a lack of supportive stroma, insufficient survival stimuli, and exposure to specific pro-apoptotic molecules, like tumor necrosis factor-related, apoptosis-inducing ligand (TRAIL) and FasL. Anti-apoptotic pathways need to be upregulated in metastatic stem cells to decrease their vulnerability and resist hostile signals from the local reactive stroma and innate immune cells [12]. PARP-1-mediated resistance to TRAIL and the additional impact of PARPs on survival and death pathways may come into play at this point [12,81].

The predisposition of certain tumors to consistently develop distant metastases in particular organs results from the selection for a metastatic phenotype in the primary tumor. If the stroma of the primary tumor contains a signaling signature, the CTCs/DTCs will be primed to thrive in a similar environment. Estrogen-induced, proto-oncogene, tyrosine–protein kinase Src (Src) signaling and a CXCL12 and IGF1-rich stroma imprint luminal breast tumors to metastasize into the bone marrow [82]. Cytoplasmic PARP-1 is associated with sustained activation of Src-mediated survival signaling, whereas PARP-1 knockdown inhibits Src in pancreatic cancer cell lines [81] (Figure 3B). On the other hand, PARP-1 suppresses CXCL12 expression by increasing the methylation of the Cxcl12 promoter DNA in rat pancreatic beta cells through the recruitment and PARylation of TET1 [36,83]. Broader epigenetic changes underlie the role of PARP-1 in tumor-associated calcium signal transducer 2 (TROP2) expressing neuroendocrine prostate cancer (NEPC) metastasis. NEPC is a particularly aggressive subtype of castration-resistant prostate cancer that usually arises in response to hormonal therapy. TROP2-driven prostate cancer xenografts are sensitive to PARP inhibition [84]. TROP2 induces the expression of PARP-1 through c-MYC. This is accompanied by DNA condensation and a decrease in histone methylation (H3K27me3) that can be reversed the PARP inhibitor, talozaparib. In a recent study, PARP inhibition in BRCA1/2-negative breast cancer orchestrated a tumor-supporting microenvironment by inactivating PARP-2. PARP-2 deficiency in the tumor leads to increased RANKL and decreased osteoprotegerin expression. These cytokines trigger osteoclast differentiation and bone resorption. Inhibition of PARP-2 in the osteoclasts results in CCL3 downregulation due to the loss of activating NF-κB, facilitating access of repressive β-catenin to the CCL3 promoter. The compromised CCL3 production, in turn, creates an immune-suppressive milieu by altering local T cell subpopulations [85]. These results raise a warning about the potentially detrimental adverse effects of PARP1/2 dual inhibitors, highlighting the importance of PARPi effects on non-cancerous, normal cells (Figure 3B).

#### Open Questions and Prospects

PARP1 generally promotes invasion and metastasis formation, due predominantly to its effect on EMT and the maintenance of a stem cell-like state. A key issue in the intersection of PARP biology and cancer biology may be the targetability of PARPs for metastasis reduction/inhibition. The current area of approved indication for PARPis already encompasses advanced stage/metastatic ovarian, breast, prostate, or pancreatic cancers. Tankyrase inhibitors, which are in development for cancer therapy, may hold promise for the inhibition of metastasis-promoting Wnt/β-catenin and Hippo signaling. Paradoxical, metastasis-enhancing effects of chemotherapy are not uncommon, and may go unrecognized among seemingly non-responding or relapsing cases [86]. PARP1/2 dual inhibitors, as all PARPis in current use are, may facilitate a tumor-supportive microenvironment and result in the growth of breast cancer bone metastases. Given the isoform polypharmacology of PARPis and the pleiotropic functions of the PARPs in essentially all cells, there is a likelihood that such undesirable outcomes might surface in other tissue contexts as well. Whether PARP1-specific inhibition offers greater safety than non-isoform specific PARP inhibition needs further investigation.

### 2.3. Tumor-Promoting Inflammation

Tumors are often compared to wounds that do not heal [87,88]. This surprising but pertinent statement reflects the dominant role of inflammation in the formation and progression of cancers. Indeed, tumors are infiltrated to varying degrees by virtually all types of inflammatory cells, including granulocytes, macrophages, myeloid-derived suppressor cells, mast cells, NK cells, and T and B lymphocytes. Although the presence of immune cells in the tumor microenvironment permits anti-tumor immune responses to develop, inflammation is now viewed as an enabling characteristic that promotes, rather than protects, cells from cancer progression. Inflammatory cells are rich sources of soluble growth factors, angiogenic factors, proteases, and reactive oxygen/nitrogen species (ROS/RNS) that boost tumor cell proliferation, improve tumor blood supply, facilitate invasion and metastatic properties, and contribute (via the mutagenic effects of ROS) to genetic evolution and the increased malignancy of cancer [89].

**Figure 4:**
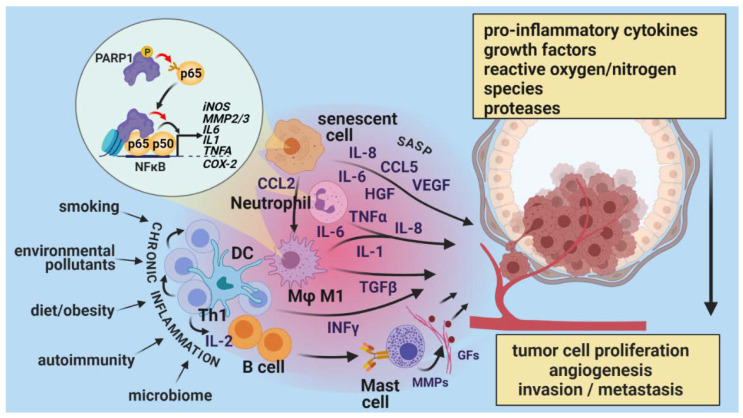
PARPs in a tumor promoting inflammation. Low-grade chronic inflammation, sustained by environmental, life-style, or disease-related factors, gives rise to elevated levels of pro-inflammatory cytokines and chemokines (IL-1, IL-6, IL-8, TNFα, etc.) produced by various types of leukocytes. These mediators are involved in the early stages of cancer development, promoting proliferation and invasion. TGF-β and interferons (IFNs) released from activated macrophages and lymphocytes promote angiogenesis and advance metastasis. The key factor underlying the inflammation-promoting role of PARP1 is its coactivator effect on NF-κB and other inflammatory transcription factors (e.g., AP-1, AP-2, YY1). ECM remodeling by MMPs produced by inflammatory cells releases sequestered growth factors, which further promote tumor growth and angiogenesis. One of the molecular markers of senescence is a senescence-associated secretory phenotype (SASP), which entails the upregulation of a variety of pro-inflammatory cytokines, growth factors, and ECM remodelers that act on other neoplastic and non-neoplastic cells in the tumor microenvironment, altering their behavior and response to chemotherapeutic drugs. The p38 MAPK and NF-κB pathways are thought to be the main choreographers of SASP [125]. PARP-1 stimulates NF-κB signaling, activated during senescence in response to various stimuli, and configures a chemokine ligand-2 (CCL2)-containing secretome that promotes tumor progression and metastasis, resulting in therapy failure. Inhibition of PARP-1 or NF-κB prevents the proinvasive properties of the secretome [126].

How do PARP enzymes affect inflammation? Many PARP family members are known to positively or negatively regulate the inflammatory response [90]. The effects of PARP-1 are clear: PARP-1 unequivocally promotes inflammation in virtually all animal models tested [91]. PARPi treatment or genetic inactivation (knockout) of PARP-1 suppresses acute and chronic inflammation in organ-specific models (colitis, experimental allergic encephalopathy, dermatitis, arthritis, hepatitis, pancreatitis, asthma, etc.) and generalized inflammation (e.g., septic, endotoxin, or flagellin shock) [6,11,92,93,94,95,96,97,98,99,100,101,102,103,104,105]. These anti-inflammatory effects are characterized by reduced inflammatory cell migration and lowered expression of inflammatory cytokines, chemokines, inducible nitric oxide synthase, adhesion molecules, and metalloproteinases. Moreover, suppression of ROS/RNS production and oxidative lipid, protein, and DNA modifications are common features of the anti-inflammatory actions of PARPi or PARP-1 knockout. The key factor underlying the inflammation-promoting role of PARP-1 is its coactivator effect on NF-κB [106,107] and other inflammatory transcription factors (e.g., AP-1, AP-2, YY1) [33,108] (Figure 4). Conversely, PARylation of SP1 by PARP-1 interferes with the binding of this anti-inflammatory transcription factors to its consensus sequence, which may suppress inflammation (a co-repressor function). Moreover, the functional consequences of PARP-1–NF-κB interactions may also be context-dependent. For example, the histone methyltransferase polycomb repressive complex 2 (PRC2) is often mutated in cancers [109]. Inhibition or knockout of PARP-1 on an inactive PRC2 background leads to NF-κB activation, increased angiogenesis, and macrophage polarization to the tumor-promoting M2 phenotype [110]. Furthermore, in immune-mediated inflammation, the immunomodulatory roles of PARPs (see below) should also be taken into account.

As for other PARP family members, PARP-9 and PARP-14 regulate macrophage polarization [111]. Macrophages are highly plastic cells and display a continuous spectrum of phenotypes, ranging from proinflammatory M1 to anti-inflammatory M2 macrophages [112]. PARP-9 and PARP-14 cross-regulate these polarization processes. PARP-9 promotes M1 polarization and PARP-14 mediates M2 polarization. PARP-14 MARylates STAT1, leading to the inhibition of STAT1 phosphorylation, whereas PARP-9 interferes with STAT1 MARylation [111]. Thus, the interplay of these two PARP enzymes regulates M1/M2 balance, a critical control point of inflammation.

While studying the molecular mechanisms underlying cherubism, a bone disease characterized by systemic inflammation, a link was identified between tankyrases 1 and 2 and tumor necrosis factor alpha (TNFα) expression [113]. The typical disease-causing mutations that occur in SH3 binding protein 2 (SH3BP2) prevent tankyrases from binding to SH3BP2, resulting in ubiquitination-mediated degradation of SH3BP2 by the E3-ubiquitin ligase RNF146. In turn, SH3BP2 stabilization triggers pathological signaling, including increased TNFα production by macrophages [113].

Moreover, PARP10 acts as a corepressor of NF-κB in response to TNFα and IL-1β. PARP-10 MARylates NF-κB essential modulator (NEMO, a subunit of the IκB kinase complex that activates NF-κB), preventing its ubiquitination [114].

A role of PARP-2 in inflammation has also been proposed [115]. The severity of chronic colitis was lower in mice treated with an antisense, oligonucleotide-targeting PARP-2 [116]. Moreover, experimental allergic encephalomyelitis (a model of multiple sclerosis) was less suppressed in PARP-2^−/−^ mice [117].

PARP-4 is also known as vPARP (vault PARP), because it was identified as an interacting partner of the major vault protein (MVP) [118]. Vaults are poorly characterized cellular ribonucleoprotein assemblies. Although PARP-4 has not been linked to inflammation, its interacting partner, MVP, regulates TNFα, IL6, and IL8 production in various cell types [119,120]. MVP can also suppress NF-κB signaling [121]. Thus, the role of MVP appears to be context-dependent and controversial. Nonetheless, PARP-4 may play a pro/anti-inflammatory role as a modulator of MVP, a possibility that is worth investigating.

PARP-7 MARylates and coactivates Liver X receptor (LXR) [122]. Since LXR agonists suppress inflammation [123], PARP-7 may play an anti-inflammatory role, a theoretical possibility awaiting experimental confirmation. PARP-12 has also been loosely linked to inflammation. PARP-12 associates with the innate signaling adaptor protein, p62, to promote NF-κB signaling [124].

#### Open Questions and Prospects

Inflammatory signaling is one of the key roles played by PARP-1. Moreover, many other PARPs have the potential to modulate (promote or inhibit) inflammation. While some molecular mechanisms underlying these pro/anti-inflammatory effects have been explored, a systematic investigation of changes in the tumor-intrinsic inflammatory state upon PARPi treatment is missing. Considering the mostly cancer-promoting effect of tumor-associated macrophages (TAMs), which have a “tumor-friendly”, M2-like phenotype, inhibitors of PARP-14 may have the potential to reverse M2 polarization of TAM, similar to models of IL-4-induced M2 polarization.

### 2.4. Evading Immune Destruction

Tumor immunology is one of the hottest topics in cancer biology; advances in tumor immunology have contributed to remarkable success stories, as represented by immune checkpoint inhibitors (anti-CTLA4, anti-PD1, and anti-PD1L antibodies) and chimeric antigen receptor (CAR) expressing engineered T cells (CAR-T). The tumor microenvironment contains many effector immune cells and immune-modulatory cell types. As for effector immune cells, CD8+ cytotoxic T lymphocytes (CTLs), natural killer (NK) cells, and Th1 type helper T cells collaborate to kill cancer cells. On the other hand, MDSCs representing immature myeloid cells, tumor-associated macrophages (polarized in the tumor to M2 phenotype), regulatory T cells (T_reg_), and granulocytes are viewed as tumors’ best friends, mitigating antitumor immune responses. A delicate balance between these two arms determines the effectiveness of anticancer immune attacks. Experiments involving the transplantation of tumors that have arisen in either immunocompetent or immunodeficient mice into immunocompetent or immunodeficient hosts suggest that tumors face immune pressure that keeps them dormant, but escape mechanisms may develop with time, triggering tumor growth [2,127,128]. An important hallmark of tumors is immune response evasion by multiple complex mechanisms. Mediators of tumor-associated immune suppression include, but are not limited to, the recruitment of immunosuppressive cells (e.g., MDSC, T_reg_), the expression of immune checkpoint proteins, production of TGF-β [129] or CCL21 [130], and induction of cell death in anti-tumor effector cells [2,131]. Under immune system pressure, a new neoplastic phenotype can be positively selected in the tumor that is resistant to anti-tumor immunity. This selection process is known as immune editing [127]. Thus, without interventions, effective anti-tumor immunity is more like a theoretical opportunity rather than a friend to rely on. While this statement may be true for established tumors that reach detection limits, the possibility cannot be excluded that the immune system eradicates incipient neoplastic cells, preventing the appearance of tumors. The idea of effective immune surveillance is supported by increased tumor formation in hosts lacking CTLs, NK cells, or Th1 cells [2,132]. Moreover, the success of recent immunotherapy approaches clearly demonstrates the power of anti-cancer immunity, if we manage to eliminate the brakes that tumors put on the immune system [133,134].

**Figure 5:**
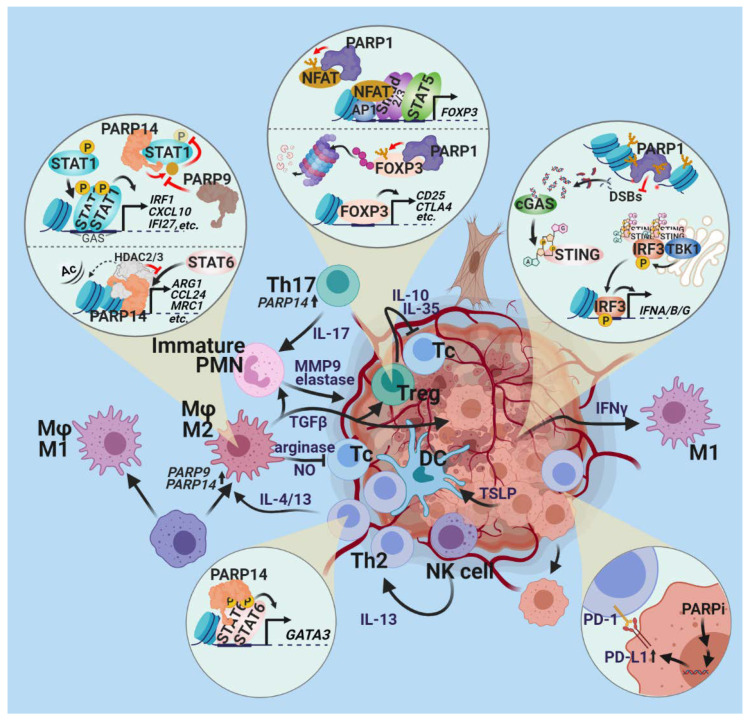
PARPs and tumor immune system evasion. The different PARP family members have variegated effects on the interaction of the immune system with the tumor. The development of regulatory T cells (T_reg_-s) is regulated by PARP1. PARP-1 PARylates nuclear factor of activated T-cells (NFAT); regulating its nuclear retention; binding to its consensus sequence; and activating genes encoding IL-2, IL-4 and and Foxp3. PARP-1 PARylates Foxp3, a master regulator of T_reg_ development impeding the development of T_reg_-s. PARP-1 also controls antitumor immune responses at the level of innate immunity. In tumor cells incurring genotoxic stress, DSBs fragment the genomic DNA. The released DNA is recognized by the cytosolic innate nucleic acid sensor cGAS. Cyclic GMP–AMP produced by cGAS activates STING and IRF-mediated IFN synthesis, leading to the inflammatory polarization of macrophages. PARP-1 and -2 counteract this process by enhancing double-strand break repair (DSBR) and limiting the occurrence of DSBs. PARP-9 and PARP-14 regulate macrophage polarization. PARP-14 MARylates STAT1, leading to inhibition of STAT1 phosphorylation, whereas PARP-9 interferes with STAT1 MARylation, cross-regulating the balance of the M1/M2 polarization of macrophages. PARP-14, acting as a promoter-bound co-regulator, stimulates STAT6-mediated GATA3 expression in T cells, directing them toward a Th2 phenotype. PARP inhibition was shown to induce the expression of the immune checkpoint regulator PD-1L in tumors.

The role of PARP enzymes in immunity is widespread, and begins with the early steps of lymphocyte development. PARP2 regulates T cell development in the thymus, as indicated by the reduced number of double-positive (CD4+ and CD8+) thymocytes in the thymus. The development of regulatory T cells (T_reg_-s) is regulated by PARP1 (Figure 5), as indicated by the increase in functional T_reg_-s in PARP1 knockout mice [135]. Moreover, PARP1 and PARP2 cooperate to repair DNA breaks in proliferating T and B lymphocytes [136,137]. Accordingly, T cell deficiency affects both CD4+ and CD8+ T cells in mice with dual, but not single, deficiency in PARP1 and PARP2 [137]. Similarly, B lymphocytes are reduced in the bone marrow and the periphery, affecting the mature, follicular B cell compartment of dual knockout animals. The role of PARP1 in immune-mediated inflammation, such as asthma [69,101,138,139] and experimental allergic encephalopathy (a model of multiple sclerosis), [140,141] also indicates the involvement of PARP1 in immunoregulation. The molecular mechanism underlying the T cell regulatory functions of PARP1 likely involves nuclear factor of activated T cells (NFAT) (Figure 5). T cell activation is accompanied by PARP1 activation in the absence of DNA breaks. Activated PARP1 interacts with and PARylates NFAT, resulting in a shortened time in the nucleus and suppression of its activation [142]. In contrast, another study found that PARP1-mediated PARylation of NFAT is required for NFAT binding to its consensus sequence and activation of genes encoding IL-2 and IL-4. Further clarification is needed concerning PARP1 activation during T cell activation and the discrepancies regarding PARP1 involvement in T cell functions. Another convergence point of T cell activation signaling pathways is Foxp3. Foxp3-positive T_reg_-s are increased in PARP-1 knockout mice compared to wild-type animals. Furthermore, PARP1 limits the immunosuppressive effects of T_reg_-s by reducing expression of the Foxp3 gene [143] and destabilizing the Foxp3 protein via PARylation [144].

PARP-1 also controls antitumor immune responses at the level of innate immunity. In macrophages, PARP-1 is required for efficient Th1 and CTL responses in colon tumor-bearing mice [145]. Moreover, the recruitment of professional antigen-presenting dendritic cells and natural killer cells to immune-mediated and viral inflammation sites depends on PARP1 [146,147,148]. However, the same effect has not yet been observed in tumor models. The modulation of macrophage polarization by PARP-14 and PARP-9 (Figure 5), as detailed in the previous chapter, may also affect the activity of anticancer immune responses via antigen presentation and Th cell activation.

#### Open Questions and Prospects

Overall, the presence of PARP-1 and PARP-2 in the tumor microenvironment appears to limit anticancer immunity and support tumor growth. This is supported by studies reporting limited tumor growth of PARP-1/PARP-2 proficient cancer cell lines in PARP-1 or PARP-2 knockout host mice [149,150]. The extent that this effect depends on PARP-1/2 enzymatic activity is unknown. A systematic investigation into the contribution of PARP enzymes to the composition of the tumor microenvironment with special regard to various anti-tumor effector and suppressor cells is needed. One of the most interesting aspects concerning the role of PARP-1 in tumor immunology was a report demonstrating upregulation of the immunosuppressive cancer cell ligand PD-L1 in breast cancer cells treated with the PARP inhibitors, olaparib, talazoparib, and rucaparib [151]. This finding triggered a series of clinical trials assessing the effectiveness of combination therapies involving PARPi and immune checkpoint inhibitors. The question of whether or not the T cell-suppressing effect of PARPis (see above) limits the effectiveness of these combinations in cancer patients is important. Several findings suggest that intra- and extracellular NAD can modulate various aspects of antitumor immunity, ranging from macrophage polarization to T_reg_ survival and function [152]. Further studies are clearly needed to unravel the complexity of mechanisms operating at the intersection of PARylation, NAD homeostasis, and anticancer immune responses.

## 3. Conclusions

Our current understanding of the complex role of PARylation enzymes in cancer cell non-intrinsic hallmarks is very limited. The most detailed picture we have is about the proinflammatory role of PARP-1. However, there are a number of unknowns, including (a) whether and how the proinflammatory effects of PARP1 impact cancer-associated inflammation; (b) whether inhibition of cancer-associated inflammation by PARPis provides therapeutic benefits, and (c) how spatiotemporal factors and tumor-specific differences modulate such potential anticancer effects. PARPs other than PARP-1 also play widespread roles in inflammation. However, none of these effects have been investigated in the context of cancer-associated inflammation. Specific targeting tools are clearly needed to address burning questions in this exciting field. One of the most promising lines of investigation is focused on the crossroads of immune checkpoint regulation and synthetic lethality by PARPi. However, the dependence of targeted anticancer cell therapy efficiency on PARPs is unknown. Based on the information discussed in this paper, the possibility that PARP/PARylation-targeted therapies may also help alleviate the most feared cancer hallmark, invasion and metastasis, cannot be excluded.

## Data Availability

No new data were created or analyzed in this study. Data sharing is not applicable to this article.

## References

[1] Hanahan D., Weinberg R.A. (2000). The hallmarks of cancer. Cell.

[2] Hanahan D., Weinberg R.A. (2011). Hallmarks of cancer: The next generation. Cell.

[3] Hottiger M.O., Hassa P.O., Lüscher B., Schüler H., Koch-Nolte F. (2010). Toward a unified nomenclature for mammalian ADP-ribosyltransferases. Trends Biochem. Sci..

[4] Hassa P.O., Hottiger M.O. (2008). The diverse biological roles of mammalian PARPS, a small but powerful family of poly-ADP-ribose polymerases. Front Biosci..

[5] Burkle A., Virag L. (2013). Poly(ADP-ribose): PARadigms and PARadoxes. Mol. Aspects Med..

[6] Hegedus C., Virag L. (2014). Inputs and outputs of poly(ADP-ribosyl)ation: Relevance to oxidative stress. Redox Biol..

[7] Robinson N., Ganesan R., Hegedus C., Kovacs K., Kufer T.A., Virag L. (2019). Programmed necrotic cell death of macrophages: Focus on pyroptosis, necroptosis, and parthanatos. Redox Biol..

[8] Virag L. (2005). The expanding universe of poly(ADP-ribosyl)ation. Cell. Mol. Life Sci..

[9] Virag L. (2013). 50 Years of poly(ADP-ribosyl)ation. Mol. Aspects Med..

[10] Virag L., Robaszkiewicz A., Rodriguez-Vargas J.M., Oliver F.J. (2013). Poly(ADP-ribose) signaling in cell death. Mol. Aspects Med..

[11] Virag L., Szabo C. (2002). The therapeutic potential of poly(ADP-ribose) polymerase inhibitors. Pharmacol. Rev..

[12] Demény M.A., Virág L. (2021). The PARP enzyme family and the hallmarks of cancer. Part 1. Cell intrinsic hallmarks. Cancers (Basel).

[13] Jayson G.C., Kerbel R., Ellis L.M., Harris A.L. (2016). Antiangiogenic therapy in oncology: Current status and future directions. Lancet.

[14] Nishida N., Yano H., Nishida T., Kamura T., Kojiro M. (2006). Angiogenesis in cancer. Vasc. Health Risk Manag..

[15] Liekens S., Schols D., Hatse S. (2010). CXCL12-CXCR4 axis in angiogenesis, metastasis and stem cell mobilization. Curr. Pharm. Des..

[16] Chu S.H., Feng D.F., Ma Y.B., Zhu Z.A., Zhang H., Qiu J.H. (2009). Stabilization of hepatocyte growth factor mRNA by hypoxia-inducible factor 1. Mol. Biol. Rep..

[17] Yu F., Lin Y., Zhan T., Chen L., Guo S. (2015). HGF expression induced by HIF-1alpha promote the proliferation and tube formation of endothelial progenitor cells. Cell Biol. Int..

[18] Rey S., Semenza G.L. (2010). Hypoxia-inducible factor-1-dependent mechanisms of vascularization and vascular remodelling. Cardiovasc. Res..

[19] Tentori L., Lacal P.M., Muzi A., Dorio A.S., Leonetti C., Scarsella M., Ruffini F., Xu W., Min W., Stoppacciaro A. (2007). Poly(ADP-ribose) polymerase (PARP) inhibition or PARP-1 gene deletion reduces angiogenesis. Eur. J. Cancer.

[20] Tentori L., Muzi A., Dorio A.S., Bultrini S., Mazzon E., Lacal P.M., Shah G.M., Zhang J., Navarra P., Nocentini G. (2008). Stable depletion of poly (ADP-ribose) polymerase-1 reduces in vivo melanoma growth and increases chemosensitivity. Eur. J. Cancer.

[21] Martínez-Romero R., Cañuelo A., Martínez-Lara E., Javier Oliver F., Cárdenas S., Siles E. (2009). Poly(ADP-ribose) polymerase-1 modulation of in vivo response of brain hypoxia-inducible factor-1 to hypoxia/reoxygenation is mediated by nitric oxide and factor inhibiting HIF. J. Neurochem..

[22] Martínez-Romero R., Martínez-Lara E., Aguilar-Quesada R., Peralta A., Oliver F.J., Siles E. (2008). PARP-1 modulates deferoxamine-induced HIF-1alpha accumulation through the regulation of nitric oxide and oxidative stress. J. Cell. Biochem..

[23] Elser M., Borsig L., Hassa P.O., Erener S., Messner S., Valovka T., Keller S., Gassmann M., Hottiger M.O. (2008). Poly(ADP-ribose) polymerase 1 promotes tumor cell survival by coactivating hypoxia-inducible factor-1-dependent gene expression. Mol. Cancer Res..

[24] Gonzalez-Flores A., Aguilar-Quesada R., Siles E., Pozo S., Rodríguez-Lara M.I., López-Jiménez L., López-Rodríguez M., Peralta-Leal A., Villar D., Martín-Oliva D. (2014). Interaction between PARP-1 and HIF-2α in the hypoxic response. Oncogene.

[25] Marti J.M., Garcia-Diaz A., Delgado-Bellido D., O’Valle F., Gonzalez-Flores A., Carlevaris O., Rodriguez-Vargas J.M., Ame J.C., Dantzer F., King G.L. (2021). Selective modulation by PARP-1 of HIF-1alpha-recruitment to chromatin during hypoxia is required for tumor adaptation to hypoxic conditions. Redox Biol..

[26] Martin-Oliva D., Aguilar-Quesada R., O’Valle F., Muñoz-Gámez J.A., Martínez-Romero R., García Del Moral R., Ruiz de Almodóvar J.M., Villuendas R., Piris M.A., Oliver F.J. (2006). Inhibition of poly(ADP-ribose) polymerase modulates tumor-related gene expression, including hypoxia-inducible factor-1 activation, during skin carcinogenesis. Cancer Res..

[27] Quiles-Perez R., Munoz-Gamez J.A., Ruiz-Extremera A., O’Valle F., Sanjuan-Nunez L., Martin-Alvarez A.B., Martin-Oliva D., Caballero T., Munoz de Rueda P., Leon J. (2010). Inhibition of poly adenosine diphosphate-ribose polymerase decreases hepatocellular carcinoma growth by modulation of tumor-related gene expression. Hepatology.

[28] Martín-Oliva D., O’Valle F., Muñoz-Gámez J.A., Valenzuela M.T., Nuñez M.I., Aguilar M., Ruiz de Almodóvar J.M., Garcia del Moral R., Oliver F.J. (2004). Crosstalk between PARP-1 and NF-kappaB modulates the promotion of skin neoplasia. Oncogene.

[29] Rajesh M., Mukhopadhyay P., Godlewski G., Bátkai S., Haskó G., Liaudet L., Pacher P. (2006). Poly(ADP-ribose)polymerase inhibition decreases angiogenesis. Biochem. Biophys. Res. Commun..

[30] Rajesh M., Mukhopadhyay P., Bátkai S., Godlewski G., Haskó G., Liaudet L., Pacher P. (2006). Pharmacological inhibition of poly(ADP-ribose) polymerase inhibits angiogenesis. Biochem. Biophys. Res. Commun..

[31] Jia J., Ye T., Cui P., Hua Q., Zeng H., Zhao D. (2016). AP-1 transcription factor mediates VEGF-induced endothelial cell migration and proliferation. Microvasc. Res..

[32] Zingarelli B., Hake P.W., O’Connor M., Denenberg A., Wong H.R., Kong S., Aronow B.J. (2004). Differential regulation of activator protein-1 and heat shock factor-1 in myocardial ischemia and reperfusion injury: Role of poly(ADP-ribose) polymerase-1. Am. J. Physiol. Heart Circ. Physiol..

[33] Andreone T.L., O’Connor M., Denenberg A., Hake P.W., Zingarelli B. (2003). Poly(ADP-ribose) polymerase-1 regulates activation of activator protein-1 in murine fibroblasts. J. Immunol..

[34] Wagner S., Hussain M.Z., Beckert S., Ghani Q.P., Weinreich J., Hunt T.K., Becker H.D., Königsrainer A. (2007). Lactate down-regulates cellular poly(ADP-ribose) formation in cultured human skin fibroblasts. Eur J. Clin. Investig..

[35] Kumar V.B., Viji R.I., Kiran M.S., Sudhakaran P.R. (2007). Endothelial cell response to lactate: Implication of PAR modification of VEGF. J. Cell. Physiol..

[36] Tolić A., Grdović N., Dinić S., Rajić J., Đorđević M., Sinadinović M., Arambašić Jovanović J., Mihailović M., Poznanović G., Uskoković A. (2019). Absence of PARP-1 affects Cxcl12 expression by increasing DNA demethylation. J. Cell. Mol. Med..

[37] Kim T.H., Hur E.G., Kang S.J., Kim J.A., Thapa D., Lee Y.M., Ku S.K., Jung Y., Kwak M.K. (2011). NRF2 blockade suppresses colon tumor angiogenesis by inhibiting hypoxia-induced activation of HIF-1α. Cancer Res..

[38] Wu T., Wang X.J., Tian W., Jaramillo M.C., Lau A., Zhang D.D. (2014). Poly(ADP-ribose) polymerase-1 modulates Nrf2-dependent transcription. Free Radic. Biol. Med..

[39] Baudino T.A., McKay C., Pendeville-Samain H., Nilsson J.A., Maclean K.H., White E.L., Davis A.C., Ihle J.N., Cleveland J.L. (2002). c-Myc is essential for vasculogenesis and angiogenesis during development and tumor progression. Genes Dev..

[40] Mostocotto C., Carbone M., Battistelli C., Ciotti A., Amati P., Maione R. (2014). Poly(ADP-ribosyl)ation is required to modulate chromatin changes at c-MYC promoter during emergence from quiescence. PLoS ONE.

[41] Zhang Y., Liu S., Mickanin C., Feng Y., Charlat O., Michaud G.A., Schirle M., Shi X., Hild M., Bauer A. (2011). RNF146 is a poly(ADP-ribose)-directed E3 ligase that regulates axin degradation and Wnt signalling. Nat. Cell Biol..

[42] Feijs K.L., Kleine H., Braczynski A., Forst A.H., Herzog N., Verheugd P., Linzen U., Kremmer E., Lüscher B. (2013). ARTD10 substrate identification on protein microarrays: Regulation of GSK3β by mono-ADP-ribosylation. Cell Commun. Signal.

[43] Rodríguez M.I., Majuelos-Melguizo J., Martí Martín-Consuegra J.M., Ruiz de Almodóvar M., López-Rivas A., Javier Oliver F. (2015). Deciphering the insights of poly(ADP-ribosylation) in tumor progression. Med. Res. Rev..

[44] Rodríguez M.I., Peralta-Leal A., O’Valle F., Rodriguez-Vargas J.M., Gonzalez-Flores A., Majuelos-Melguizo J., López L., Serrano S., de Herreros A.G., Rodríguez-Manzaneque J.C. (2013). PARP-1 regulates metastatic melanoma through modulation of vimentin-induced malignant transformation. PLoS Genet..

[45] McPhee T.R., McDonald P.C., Oloumi A., Dedhar S. (2008). Integrin-linked kinase regulates E-cadherin expression through PARP-1. Dev. Dyn..

[46] Rodríguez M.I., González-Flores A., Dantzer F., Collard J., de Herreros A.G., Oliver F.J. (2011). Poly(ADP-ribose)-dependent regulation of Snail1 protein stability. Oncogene.

[47] Lönn P., van der Heide L.P., Dahl M., Hellman U., Heldin C.H., Moustakas A. (2010). PARP-1 attenuates Smad-mediated transcription. Mol. Cell.

[48] Dahl M., Maturi V., Lönn P., Papoutsoglou P., Zieba A., Vanlandewijck M., van der Heide L.P., Watanabe Y., Söderberg O., Hottiger M.O. (2014). Fine-tuning of Smad protein function by poly(ADP-ribose) polymerases and poly(ADP-ribose) glycohydrolase during transforming growth factor β signaling. PLoS ONE.

[49] Lambert A.W., Pattabiraman D.R., Weinberg R.A. (2017). Emerging Biol.ogical Principles of Metastasis. Cell.

[50] Polyak K., Weinberg R.A. (2009). Transitions between epithelial and mesenchymal states: Acquisition of malignant and stem cell traits. Nat. Rev. Cancer.

[51] Nieto M.A. (2013). Epithelial plasticity: A common theme in embryonic and cancer cells. Science.

[52] Overholtzer M., Zhang J., Smolen G.A., Muir B., Li W., Sgroi D.C., Deng C.X., Brugge J.S., Haber D.A. (2006). Transforming properties of YAP, a candidate oncogene on the chromosome 11q22 amplicon. Proc. Natl. Acad. Sci. USA.

[53] Lamar J.M., Stern P., Liu H., Schindler J.W., Jiang Z.G., Hynes R.O. (2012). The Hippo pathway target, YAP, promotes metastasis through its TEAD-interaction domain. Proc. Natl. Acad. Sci. USA.

[54] Zhao B., Wei X., Li W., Udan R.S., Yang Q., Kim J., Xie J., Ikenoue T., Yu J., Li L. (2007). Inactivation of YAP oncoprotein by the Hippo pathway is involved in cell contact inhibition and tissue growth control. Genes Dev..

[55] Zhao B., Li L., Lu Q., Wang L.H., Liu C.Y., Lei Q., Guan K.L. (2011). Angiomotin is a novel Hippo pathway component that inhibits YAP oncoprotein. Genes Dev..

[56] Wang W., Li N., Li X., Tran M.K., Han X., Chen J. (2015). Tankyrase Inhibitors Target YAP by Stabilizing Angiomotin Family Proteins. Cell Rep..

[57] Wu X., Chen H., Parker B., Rubin E., Zhu T., Lee J.S., Argani P., Sukumar S. (2006). HOXB7, a homeodomain protein, is overexpressed in breast cancer and confers epithelial-mesenchymal transition. Cancer Res..

[58] Wu X., Ellmann S., Rubin E., Gil M., Jin K., Han L., Chen H., Kwon E.M., Guo J., Ha H.C. (2012). ADP ribosylation by PARP-1 suppresses HOXB7 transcriptional activity. PLoS ONE.

[59] Pu H., Horbinski C., Hensley P.J., Matuszak E.A., Atkinson T., Kyprianou N. (2014). PARP-1 regulates epithelial-mesenchymal transition (EMT) in prostate tumorigenesis. Carcinogenesis.

[60] Karicheva O., Rodriguez-Vargas J.M., Wadier N., Martin-Hernandez K., Vauchelles R., Magroun N., Tissier A., Schreiber V., Dantzer F. (2016). PARP3 controls TGFβ and ROS driven epithelial-to-mesenchymal transition and stemness by stimulating a TG2-Snail-E-cadherin axis. Oncotarget.

[61] Navas T., Kinders R.J., Lawrence S.M., Ferry-Galow K.V., Borgel S., Hollingshead M.G., Srivastava A.K., Alcoser S.Y., Makhlouf H.R., Chuaqui R. (2020). Clinical Evolution of Epithelial-Mesenchymal Transition in Human Carcinomas. Cancer Res..

[62] Ordonez L.D., Hay T., McEwen R., Polanska U.M., Hughes A., Delpuech O., Cadogan E., Powell S., Dry J., Tornillo G. (2019). Rapid activation of epithelial-mesenchymal transition drives PARP inhibitor resistance in Brca2-mutant mammary tumours. Oncotarget.

[63] Shay G., Lynch C.C., Fingleton B. (2015). Moving targets: Emerging roles for MMPs in cancer progression and metastasis. Matrix Biol..

[64] Yan B., Jiang Z., Cheng L., Chen K., Zhou C., Sun L., Qian W., Li J., Cao J., Xu Q. (2018). Paracrine HGF/c-MET enhances the stem cell-like potential and glycolysis of pancreatic cancer cells via activation of YAP/HIF-1α. Exp. Cell Res..

[65] Matsui S., Osada S., Tomita H., Komori S., Mori R., Sanada Y., Takahashi T., Yamaguchi K., Yoshida K. (2010). Clinical significance of aggressive hepatectomy for colorectal liver metastasis, evaluated from the HGF/c-Met pathway. Int. J. Oncol..

[66] Mariani M., McHugh M., Petrillo M., Sieber S., He S., Andreoli M., Wu Z., Fiedler P., Scambia G., Shahabi S. (2014). HGF/c-Met axis drives cancer aggressiveness in the neo-adjuvant setting of ovarian cancer. Oncotarget.

[67] Wei W., Lv S., Zhang C., Tian Y. (2018). Potential role of HGF-PARP-1 signaling in invasion of ovarian cancer cells. Int. J. Clin. Exp. Pathol..

[68] Mishra M., Kowluru R.A. (2017). Role of PARP-1 as a novel transcriptional regulator of MMP-9 in diabetic retinopathy. Biochim. Biophys. Acta Mol. Basis Dis..

[69] Virág L. (2005). Poly(ADP-ribosyl)ation in asthma and other lung diseases. Pharmacol. Res..

[70] Ghorai A., Sarma A., Chowdhury P., Ghosh U. (2016). PARP-1 depletion in combination with carbon ion exposure significantly reduces MMPs activity and overall increases TIMPs expression in cultured HeLa cells. Radiat. Oncol..

[71] El-Hamoly T., Hegedűs C., Lakatos P., Kovács K., Bai P., El-Ghazaly M.A., El-Denshary E.S., Szabó É., Virág L. (2014). Activation of poly(ADP-ribose) polymerase-1 delays wound healing by regulating keratinocyte migration and production of inflammatory mediators. Mol. Med..

[72] Nicolescu A.C., Holt A., Kandasamy A.D., Pacher P., Schulz R. (2009). Inhibition of matrix metalloproteinase-2 by PARP inhibitors. Biochem. Biophys. Res. Commun..

[73] Li Q., Li M., Wang Y.L., Fauzee N.J., Yang Y., Pan J., Yang L., Lazar A. (2012). RNA interference of PARG could inhibit the metastatic potency of colon carcinoma cells via PI3-kinase/Akt pathway. Cell. Physiol. Biochem..

[74] Kim J., Yum S., Kang C., Kang S.-J. (2016). Gene-gene interactions in gastrointestinal cancer susceptibility. Oncotarget.

[75] Kim J., Pyun J.A., Cho S.W., Lee K., Kwack K. (2011). Lymph node metastasis of gastric cancer is associated with the interaction between poly (ADP-ribose) polymerase 1 and matrix metallopeptidase 2. DNA Cell Biol..

[76] Schlesinger M. (2018). Role of platelets and platelet receptors in cancer metastasis. J. Hematol. Oncol..

[77] Placke T., Kopp H.G., Salih H.R. (2012). The wolf in sheep’s clothing: Platelet-derived "pseudo self" impairs cancer cell "missing self" recognition by NK cells. Oncoimmunology.

[78] Zingarelli B., Salzman A.L., Szabó C. (1998). Genetic disruption of poly (ADP-ribose) synthetase inhibits the expression of P-selectin and intercellular adhesion molecule-1 in myocardial ischemia/reperfusion injury. Circ. Res..

[79] Yan J., Li M., Threadgill M.D., Wang Y., Fu W. (2013). Decreasing P-selectin and ICAM-1 via activating Akt: A possible mechanism by which PARG inhibits adhesion of mouse colorectal carcinoma CT26 cells to platelets. Cancer Gene Ther..

[80] Dutta P., Paico K., Gomez G., Wu Y., Vadgama J.V. (2020). Transcriptional Regulation of CCL2 by PARP1 Is a Driver for Invasiveness in Breast Cancer. Cancers (Basel).

[81] Xu F., Sun Y., Yang S.Z., Zhou T., Jhala N., McDonald J., Chen Y. (2019). Cytoplasmic PARP-1 promotes pancreatic cancer tumorigenesis and resistance. Int. J. Cancer.

[82] Zhang X.H., Wang Q., Gerald W., Hudis C.A., Norton L., Smid M., Foekens J.A., Massagué J. (2009). Latent bone metastasis in breast cancer tied to Src-dependent survival signals. Cancer Cell.

[83] Marković J., Grdović N., Dinić S., Karan-Djurašević T., Uskoković A., Arambašić J., Mihailović M., Pavlović S., Poznanović G., Vidaković M. (2013). PARP-1 and YY1 are important novel regulators of CXCL12 gene transcription in rat pancreatic beta cells. PLoS ONE.

[84] Hsu E.C., Rice M.A., Bermudez A., Marques F.J.G., Aslan M., Liu S., Ghoochani A., Zhang C.A., Chen Y.S., Zlitni A. (2020). Trop2 is a driver of metastatic prostate cancer with neuroendocrine phenotype via PARP1. Proc. Natl. Acad. Sci. USA.

[85] Zuo H., Yang D., Yang Q., Tang H., Fu Y.X., Wan Y. (2020). Differential regulation of breast cancer bone metastasis by PARP1 and PARP2. Nat. Commun..

[86] D’Alterio C., Scala S., Sozzi G., Roz L., Bertolini G. (2020). Paradoxical effects of chemotherapy on tumor relapse and metastasis promotion. Semin. Cancer Biol..

[87] Dvorak H.F. (1986). Tumors: Wounds that do not heal. Similarities between tumor stroma generation and wound healing. N. Engl. J. Med..

[88] Schäfer M., Werner S. (2008). Cancer as an overhealing wound: An old hypothesis revisited. Nat. Rev. Mol. Cell Biol..

[89] Hegedus C., Kovacs K., Polgar Z., Regdon Z., Szabo E., Robaszkiewicz A., Forman H.J., Martner A., Virag L. (2018). Redox control of cancer cell destruction. Redox Biol..

[90] Brady P.N., Goel A., Johnson M.A. (2019). Poly(ADP-Ribose) Polymerases in Host-Pathogen Interactions, Inflammation, and Immunity. Microbiol. Mol. Biol. Rev..

[91] Bai P., Virag L. (2012). Role of poly(ADP-ribose) polymerases in the regulation of inflammatory processes. Febs Lett..

[92] Goldfarb R.D., Marton A., Szabo E., Virag L., Salzman A.L., Glock D., Akhter I., McCarthy R., Parrillo J.E., Szabo C. (2002). Protective effect of a novel, potent inhibitor of poly(adenosine 5′-diphosphate-ribose) synthetase in a porcine model of severe bacterial sepsis. Crit. Care Med..

[93] Liaudet L., Soriano F.G., Szabo E., Virag L., Mabley J.G., Salzman A.L., Szabo C. (2000). Protection against hemorrhagic shock in mice genetically deficient in poly(ADP-ribose)polymerase. Proc. Natl. Acad. Sci. USA.

[94] Mabley J., Virag L., Jagtap P., Szabo E., Prendergast R., Perretti M., Getting S., Szabo C. (2001). Effect of a novel poly (ADP-ribose) polymerase (PARP) inhibitor in rodent models of local inflammation. Faseb J..

[95] Pacher P., Liaudet L., Mabley J.G., Soriano F.G., Virag L., Szabo C. (2002). Activation of poly(ADP-ribose) polymerase-1 is a central mechanism of lipopolysaccharide-induced acute lung inflammation. Faseb J..

[96] Mabley J.G., Jagtap P., Perretti M., Getting S.J., Salzman A.L., Virag L., Szabo E., Soriano F.G., Liaudet L., Abdelkarim G.E. (2001). Anti-inflammatory effects of a novel, potent inhibitor of poly (ADP-ribose) polymerase. Inflamm. Res..

[97] Marton A., Szabo E., Virag L., Salzman A., Glock D., McCarthy R., Parrillo J.E., Sabo C., Goldfarb R.D. (2001). Protective effect of a novel inhibitor of poly (ADP-ribose) synthetase in a porcine model of peritonitis. Faseb J..

[98] Liaudet L., Pacher P., Mabley J.G., Virag L., Soriano F.G., Hasko G., Szabo C. (2002). Activation of poly(ADP-ribose) polymerase-1 is a central mechanism of lipopolysaccharide-induced acute lung inflammation. Am. J. Resp. Crit. Care.

[99] Bakondi E., Bai P., Bak I., Szabo E., Gergely P., Szabo C., Virag L. (2002). Role of poly(ADP-ribose) polymerase in contact hypersensitivity. Faseb J..

[100] Virag L., Szabo E. (2011). Complex Role of Poly(Adp-Ribosyl)Ation in Shock and Other Oxidative Stress-Related Pathologies. Shock.

[101] Virag L., Bai P., Bak I., Pacher P., Mabley J., Liaudet L., Bakondl E., Gergely P., Kollai M., Szabo C. (2004). Effects of poly(ADP-ribose) polymerase inhibition on inflammatory cell migration in a murine model of asthma. Med. Sci. Monitor.

[102] Soriano F.G., Liaudet L., Szabo E., Virag L., Mabley J.G., Pacher P., Szabo C. (2002). Resistance to acute septic peritonitis in poly(ADP-ribose) polymerase-1 deficient mice. Faseb J..

[103] Scott G.S., Hake P., Kean R.B., Virag L., Szabo C., Hooper D.C. (2001). Role of poly(ADP-ribose) synthetase activation in the development of experimental allergic encephalomyelitis. J. Neuroimmunol..

[104] Szabo E., Bodnar E., Erdelyi K., Hegedus C., Bakondi E., Remenyik E., Virag L. (2009). Possible role of the nitric oxide-peroxynitrite-poly(ADP-ribose) polymerase pathway in chronic wounds. J. Investig. Dermatol..

[105] El-Hamoly T., Hajnady Z., Nagy-Penzes M., Bakondi E., Regdon Z., Demeny M.A., Kovacs K., Hegedus C., Abd El-Rahman S.S., Szabo E. (2021). Poly(ADP-Ribose) Polymerase 1 Promotes Inflammation and Fibrosis in a Mouse Model of Chronic Pancreatitis. Int. J. Mol. Sci..

[106] Hassa P.O., Hottiger M.O. (2002). The functional role of poly(ADP-ribose)polymerase 1 as novel coactivator of NF-kappaB in inflammatory disorders. Cell. Mol. Life Sci..

[107] Oliver F.J., Ménissier-de Murcia J., Nacci C., Decker P., Andriantsitohaina R., Muller S., de la Rubia G., Stoclet J.C., de Murcia G. (1999). Resistance to endotoxic shock as a consequence of defective NF-kappaB activation in poly (ADP-ribose) polymerase-1 deficient mice. Embo J..

[108] Oei S.L., Shi Y. (2001). Poly(ADP-ribosyl)ation of transcription factor Yin Yang 1 under conditions of DNA damage. Biochem. Biophys. Res. Commun..

[109] Laugesen A., Hojfeldt J.W., Helin K. (2016). Role of the Polycomb Repressive Complex 2 (PRC2) in Transcriptional Regulation and Cancer. Cold Spring Harb. Perspect. Med..

[110] Yang A.Y., Choi E.B., So Park M., Kim S.K., Park M.S., Kim M.Y. (2020). PARP1 and PRC2 double deficiency promotes BRCA-proficient breast cancer growth by modification of the tumor microenvironment. Febs J..

[111] Iwata H., Goettsch C., Sharma A., Ricchiuto P., Goh W.W., Halu A., Yamada I., Yoshida H., Hara T., Wei M. (2016). PARP9 and PARP14 cross-regulate macrophage activation via STAT1 ADP-ribosylation. Nat. Commun..

[112] Locati M., Curtale G., Mantovani A. (2020). Diversity, Mechanisms, and Significance of Macrophage Plasticity. Annu. Rev. Pathol..

[113] Levaot N., Voytyuk O., Dimitriou I., Sircoulomb F., Chandrakumar A., Deckert M., Krzyzanowski P.M., Scotter A., Gu S., Janmohamed S. (2011). Loss of Tankyrase-mediated destruction of 3BP2 is the underlying pathogenic mechanism of cherubism. Cell.

[114] Verheugd P., Forst A.H., Milke L., Herzog N., Feijs K.L., Kremmer E., Kleine H., Luscher B. (2013). Regulation of NF-kappaB signalling by the mono-ADP-ribosyltransferase ARTD10. Nat. Commun..

[115] Ali S.O., Khan F.A., Galindo-Campos M.A., Yelamos J. (2016). Understanding specific functions of PARP-2: New lessons for cancer therapy. Am. J. Cancer Res..

[116] Popoff I., Jijon H., Monia B., Tavernini M., Ma M., McKay R., Madsen K. (2002). Antisense oligonucleotides to poly(ADP-ribose) polymerase-2 ameliorate colitis in interleukin-10-deficient mice. J. Pharmacol. Exp. Ther..

[117] Kamboj A., Lu P., Cossoy M.B., Stobart J.L., Dolhun B.A., Kauppinen T.M., de Murcia G., Anderson C.M. (2013). Poly(ADP-ribose) polymerase 2 contributes to neuroinflammation and neurological dysfunction in mouse experimental autoimmune encephalomyelitis. J. Neuroinflamm..

[118] Kickhoefer V.A., Siva A.C., Kedersha N.L., Inman E.M., Ruland C., Streuli M., Rome L.H. (1999). The 193-kD vault protein, VPARP, is a novel poly(ADP-ribose) polymerase. J. Cell Biol..

[119] Peng N., Liu S., Xia Z., Ren S., Feng J., Jing M., Gao X., Wiemer E.A., Zhu Y. (2016). Inducible Major Vault Protein Plays a Pivotal Role in Double-Stranded RNA- or Virus-Induced Proinflammatory Response. J. Immunol..

[120] Ben J., Zhang Y., Zhou R., Zhang H., Zhu X., Li X., Zhang H., Li N., Zhou X., Bai H. (2013). Major vault protein regulates class A scavenger receptor-mediated tumor necrosis factor-α synthesis and apoptosis in macrophages. J. Biol. Chem..

[121] Ben J., Jiang B., Wang D., Liu Q., Zhang Y., Qi Y., Tong X., Chen L., Liu X., Zhang Y. (2019). Major vault protein suppresses obesity and atherosclerosis through inhibiting IKK-NF-κB signaling mediated inflammation. Nat. Commun..

[122] Bindesbøll C., Tan S., Bott D., Cho T., Tamblyn L., MacPherson L., Grønning-Wang L., Nebb H.I., Matthews J. (2016). TCDD-inducible poly-ADP-ribose polymerase (TIPARP/PARP7) mono-ADP-ribosylates and co-activates liver X receptors. Biochem. J..

[123] Fowler A.J., Sheu M.Y., Schmuth M., Kao J., Fluhr J.W., Rhein L., Collins J.L., Willson T.M., Mangelsdorf D.J., Elias P.M. (2003). Liver X receptor activators display anti-inflammatory activity in irritant and allergic contact dermatitis models: Liver-X-receptor-specific inhibition of inflammation and primary cytokine production. J. Investig. Dermatol..

[124] Welsby I., Hutin D., Gueydan C., Kruys V., Rongvaux A., Leo O. (2014). PARP12, an interferon-stimulated gene involved in the control of protein translation and inflammation. J. Biol. Chem..

[125] Salminen A., Kauppinen A., Kaarniranta K. (2012). Emerging role of NF-kappaB signaling in the induction of senescence-associated secretory phenotype (SASP). Cell Signal.

[126] Ohanna M., Giuliano S., Bonet C., Imbert V., Hofman V., Zangari J., Bille K., Robert C., Bressac-de Paillerets B., Hofman P. (2011). Senescent cells develop a PARP-1 and nuclear factor-{kappa}B-associated secretome (PNAS). Genes Dev..

[127] Schreiber R.D., Old L.J., Smyth M.J. (2011). Cancer immunoediting: Integrating immunity’s roles in cancer suppression and promotion. Science.

[128] Svane I.M., Engel A.M., Nielsen M.B., Ljunggren H.G., Rygaard J., Werdelin O. (1996). Chemically induced sarcomas from nude mice are more immunogenic than similar sarcomas from congenic normal mice. Eur. J. Immunol..

[129] Yang L., Pang Y., Moses H.L. (2010). TGF-beta and immune cells: An important regulatory axis in the tumor microenvironment and progression. Trends Immunol..

[130] Shields J.D., Kourtis I.C., Tomei A.A., Roberts J.M., Swartz M.A. (2010). Induction of lymphoidlike stroma and immune escape by tumors that express the chemokine CCL21. Science.

[131] Arrieta V.A., Cacho-Díaz B., Zhao J., Rabadan R., Chen L., Sonabend A.M. (2018). The possibility of cancer immune editing in gliomas. A critical review. Oncoimmunology.

[132] Waldhauer I., Steinle A. (2008). NK cells and cancer immunosurveillance. Oncogene.

[133] Sharma P., Allison J.P. (2015). Immune checkpoint targeting in cancer therapy: Toward combination strategies with curative potential. Cell.

[134] Chamoto K., Hatae R., Honjo T. (2020). Current issues and perspectives in PD-1 blockade cancer immunotherapy. Int. J. Clin. Oncol..

[135] Nasta F., Laudisi F., Sambucci M., Rosado M.M., Pioli C. (2010). Increased Foxp3+ regulatory T cells in poly(ADP-Ribose) polymerase-1 deficiency. J. Immunol..

[136] Galindo-Campos M.A., Bedora-Faure M., Farres J., Lescale C., Moreno-Lama L., Martinez C., Martin-Caballero J., Ampurdanes C., Aparicio P., Dantzer F. (2019). Coordinated signals from the DNA repair enzymes PARP-1 and PARP-2 promotes B-cell development and function. Cell Death Differ..

[137] Navarro J., Gozalbo-Lopez B., Mendez A.C., Dantzer F., Schreiber V., Martinez C., Arana D.M., Farres J., Revilla-Nuin B., Bueno M.F. (2017). PARP-1/PARP-2 double deficiency in mouse T cells results in faulty immune responses and T lymphomas. Sci. Rep..

[138] Szabó É., Kovács I., Grune T., Haczku A., Virág L. (2011). PARP-1: A new player in the asthma field?. Allergy.

[139] Ghonim M.A., Pyakurel K., Ibba S.V., Al-Khami A.A., Wang J., Rodriguez P., Rady H.F., El-Bahrawy A.H., Lammi M.R., Mansy M.S. (2015). PARP inhibition by olaparib or gene knockout blocks asthma-like manifestation in mice by modulating CD4(+) T cell function. J. Transl. Med..

[140] Scott G.S., Kean R.B., Mikheeva T., Fabis M.J., Mabley J.G., Szabó C., Hooper D.C. (2004). The therapeutic effects of PJ34 [N-(6-oxo-5,6-dihydrophenanthridin-2-yl)-N,N-dimethylacetamide.HCl], a selective inhibitor of poly(ADP-ribose) polymerase, in experimental allergic encephalomyelitis are associated with immunomodulation. J. Pharmacol. Exp. Ther..

[141] Chiarugi A. (2002). Inhibitors of poly(ADP-ribose) polymerase-1 suppress transcriptional activation in lymphocytes and ameliorate autoimmune encephalomyelitis in rats. Br. J. Pharmacol..

[142] Valdor R., Schreiber V., Saenz L., Martínez T., Muñoz-Suano A., Dominguez-Villar M., Ramírez P., Parrilla P., Aguado E., García-Cózar F. (2008). Regulation of NFAT by poly(ADP-ribose) polymerase activity in T cells. Mol. Immunol..

[143] Zhang P., Maruyama T., Konkel J.E., Abbatiello B., Zamarron B., Wang Z.Q., Chen W. (2013). PARP-1 controls immunosuppressive function of regulatory T cells by destabilizing Foxp3. PLoS ONE.

[144] Luo X., Nie J., Wang S., Chen Z., Chen W., Li D., Hu H., Li B. (2016). Poly(ADP-ribosyl)ation of FOXP3 protein mediated by PARP-1 regulates the function of regulatory T cells. J. Biol. Chem..

[145] Kunze F.A., Bauer M., Komuczki J., Lanzinger M., Gunasekera K., Hopp A.K., Lehmann M., Becher B., Müller A., Hottiger M.O. (2019). ARTD1 in Myeloid Cells Controls the IL-12/18-IFN-γ Axis in a Model of Sterile Sepsis, Chronic Bacterial Infection, and Cancer. J. Immunol..

[146] Shou Q., Fu H., Huang X., Yang Y. (2019). PARP-1 controls NK cell recruitment to the site of viral infection. JCI Insight.

[147] Echeverri Tirado L.C., Ghonim M.A., Wang J., Al-Khami A.A., Wyczechowska D., Luu H.H., Kim H., Sanchez-Pino M.D., Yélamos J., Yassin L.M. (2019). PARP-1 Is Critical for Recruitment of Dendritic Cells to the Lung in a Mouse Model of Asthma but Dispensable for Their Differentiation and Function. Mediators Inflamm..

[148] Cavone L., Aldinucci A., Ballerini C., Biagioli T., Moroni F., Chiarugi A. (2011). PARP-1 inhibition prevents CNS migration of dendritic cells during EAE, suppressing the encephalitogenic response and relapse severity. Mult. Scler..

[149] Moreno-Lama L., Galindo-Campos M.A., Martínez C., Comerma L., Vazquez I., Vernet-Tomas M., Ampurdanés C., Lutfi N., Martin-Caballero J., Dantzer F. (2020). Coordinated signals from PARP-1 and PARP-2 are required to establish a proper T cell immune response to breast tumors in mice. Oncogene.

[150] Chacon-Cabrera A., Fermoselle C., Salmela I., Yelamos J., Barreiro E. (2015). MicroRNA expression and protein acetylation pattern in respiratory and limb muscles of Parp-1(-/-) and Parp-2(-/-) mice with lung cancer cachexia. Biochim. Biophys. Acta.

[151] Jiao S., Xia W., Yamaguchi H., Wei Y., Chen M.K., Hsu J.M., Hsu J.L., Yu W.H., Du Y., Lee H.H. (2017). PARP Inhibitor Upregulates PD-L1 Expression and Enhances Cancer-Associated Immunosuppression. Clin. Cancer Res..

[152] Navas L.E., Carnero A. (2021). NAD(+) metabolism, stemness, the immune response, and cancer. Signal Transduct. Target Ther..

